# Effects of local and remote ischemic postconditioning methods on ischemiareperfusion injury in a young animal model of acute mesenteric ischemia

**DOI:** 10.1590/acb381323

**Published:** 2023-06-02

**Authors:** Mateus Souza Abreu, Ana Cristina Aoun Tannuri, Rafael Felipe Gonçalves Rodrigues, Rafael José da Silva, Josiane de Oliveira Gonçalves, Suellen Serafini, Uenis Tannuri

**Affiliations:** 1 Universidade de São Paulo – Faculdade de Medicina – Divisão de Cirurgia Pediátrica – São Paulo (SP), Brazil.

**Keywords:** Ischemia, Reperfusion Injury, Ischemic Postconditioning, Antioxidants, Rats

## Abstract

**Purpose:**

Acute mesenteric ischemia (AMI) is a condition in pediatric surgery that ranges from intestine necrosis to death. Ischemic postconditioning (IPoC) methods were developed to reduce the damage caused by revascularization. This study aimed to evaluate the efficacy of these methods in an experimental weaning rat model.

**Methods:**

Thirty-two 21-day-old Wistar rats were allocated into four groups according to the surgical procedure performed: control, ischemia-reperfusion injury (IRI), local (LIPoC) and remote IPoC (RIPoC). At euthanasia, fragments of the intestine, liver, lungs, and kidneys were submitted to histological, histomorphometric, and molecular analyses.

**Results:**

In the duodenum, intestines, and kidneys histological alterations promoted by IRI were reversed by remote postconditioning method. In the distal ileum, the histomorphometric alterations could be reversed by the postconditioning methods with more evident effects promoted by the remote method. The molecular analysis found that the levels of expression of Bax (proapoptotic) and Bcl-XL (antiapoptotic) genes in the intestine were increased by IRI. These alterations were equally reversed by the postconditioning methods, with more evident effects of the remote method.

**Conclusions:**

IPoC methods positively reduced the damage caused by IRI in weaning rats.

## Introduction

Acute mesenteric ischemia (AMI) is a potentially fatal vascular emergency, which requires early diagnosis and intervention to prevent intestinal necrosis. AMI may be associated with several conditions, among them intestinal volvulus due to intestinal malrotation, IgA vasculitis, severe congenital cardiac diseases, systemic infection, severe dehydration, familial dysautonomia, Addison disease, burnings, chemotherapy, abdominal situs inversus, fibromuscular dysplasia, compartmental abdominal syndrome, Burkitt lymphoma and dermatomyositis^1^.

In 1986, Parks and Granger^2^ demonstrated the harmful effects of reactive oxygen species produced during post-ischemic intestinal reperfusion, evidencing that ischemia-reperfusion injury (IRI) is more damaging than ischemia alone, something unknown until then. Nevertheless, Murry et al.^3^ demonstrated that producing short periods of coronary occlusion, followed by short periods of reperfusion before the main myocardial ischemia, reduced the ischemic injury in the myocardium of dogs, thus introducing the concept of ischemic preconditioning procedure (IPP).

However, there are multiple situations in which ischemia is identified after the occurrence of the injuries, making the clinical use of IPP infeasible. Taking it into consideration, in 2003, Zhao et al.^4^ presented the concept of ischemic postconditioning (IPoC), which consists of one or more short cycles of ischemia and reperfusion immediately after an important ischemia before performing permanent reperfusion. The authors showed that IPoC is as effective as IPP to prevent IRI. Following the same rationale used for IPP, there are two methods, local (LIPoC) and remote IPoC (RIPoC).

The intestine is the most sensitive organ to IRI^4^. Many authors have demonstrated the efficacy of IPoC in mesenteric IRI in adult animals^5^^-^ 9. However, IPoC efficacy is not well established in young animals, which are the most appropriate to evaluate the clinical applicability in the pediatric and neonatal population. Therefore, the aim of this study is to assess and compare LIPoC and RIPoC techniques in situation of IRI, as well as to compare both techniques, in the context of AMI in weaning Wistar rats.

## Methods

The animals were cared for according to the criteria outlined in the *Guide for the Care and Use of Laboratory Animals* prepared by the National Academy of Sciences. The study protocol was reviewed and approved by the Animal Ethics Committee at the institution (Process number 1324/2019; Faculdade de Medicina, Universidade de São Paulo, São Paulo, Brazil).

### Surgical procedures

Thirty-two 21-day-old Wistar rats (weight: 50–80 g) were allocated into four groups according to the surgical procedure. All animals were anesthetized with inhaled isoflurane until sedation, followed by intraperitoneal doses of 30 mg/kg of ketamine hydrochloride (Ketalar, Pfizer) and 10 mg/kg of dexmedetomidine (Precedex, Wyeth). Animals of control group (C) were submitted to median laparotomy, with viscera exposure. Animals of IRI group were submitted to 20 min of IRI with occlusion of the superior mesenteric vessels. Animals of LIPoC group were submitted to 6 cycles of LIPoC of 10 s of ischemia followed by 10 s of reperfusion each, after 20 min of IRI. Finally, animals of RIPoC group were submitted to right inguinotomy, with 3 cycles of 5 min of occlusion and 5 min of reperfusion of the right femoral artery, after 20 min of mesenteric IRI. The abdomen and the inguinal region were closed with Mononylon 4.0 continuous suture, and, after cleaning, the animals started postoperative recovery.

During the 24-h interval, the animals received analgesia via intraperitoneal doses of 40 mg/kg Tramadol every 8 h. At the end of this period, all animals were again anesthetized and submitted to median laparotomy for removing fragments of the intestine, liver, kidney, and lung. For an accurate analysis of the bowel, four samples were collected: one from the duodenum, one from the jejunum, and two from the ileum, proximal and distal (10 and 2 cm upstream ileocecal valve, respectively). After the biopsies, all animals were submitted to euthanasia, by deepening of anesthesia with isoflurane until cardiorespiratory arrest.

### Histological and histomorphometric analyses

For histological analysis, the samples were kept for 24 h in 10% formaldehyde. After fixation, the material was submitted to dehydration followed by paraffin embedding, and 4 μm thick histological sections were stained with hematoxylin-eosin, for general morphology studies.

The following scores were used for organ evaluation: Chiu score was used for intestinal evaluation^10^, Scheuer score for hepatic evaluation^11^, ventilator-induced lung injury score for pulmonary evaluation^12^, and a modified Banff score for renal evaluation^13^.

The histomorphometric analysis consisted of measuring the height and the diameter of the villi, the depth of crypts, the thickness of lamina propria, and the thickness of the muscle layer of the intestine.

The samples were analyzed with a Nikon Eclipse 50i optic microscope. Ten fields per sample were randomly selected and analyzed with a DS-Fi1 camera, using NIS-Elements software version 4.6 to acquire images. The different variables were quantified, and the results were transferred to an electronic spreadsheet, showing an average of the measures.

### Gene expression analysis

Real-time quantitative polymerase chain reaction (qRT-PCR) was used to assess the genes related to apoptosis: Bax (proapoptotic gene) and Bcl-XL (antiapoptotic gene). The B-actin gene was amplified as an internal control for the transcription of RNA and as a factor of correction of the quantity of cDNA synthesized. The technique utilized is based on the fluorescence monitoring of DNA amplification within each cycle. The number of copies of the gene was determined using Sybr Green I, a fluorescent dye that intersperses with double-stranded DNA. Fluorescence is captured by the thermocycler at each new PCR cycle reaction and allows the equipment to draw an amplification curve for each sample. The analysis of gene amplification conducted by qRT-PCR was performed using relative quantification, in which the gene expression of one sample is described in relation to another^14^.

### Statistical analysis

Before applying the statistical analysis intended to enable data interpretation, the Shapiro–Wilk test was performed to verify data distribution and to evaluate which is the most adequate test for each situation.

The data which presented parametric distribution were submitted to one way analysis of variance followed by the Tukey’s test. Assuming the asymmetric distribution, the data were submitted to the nonparametric Kruskal–Wallis test followed by the Dunn test. The equality hypothesis was rejected if p ≤ 0.05. All calculations were performed by SPSS software for Windows, version 18.0.

## Results

**Figure 1:**
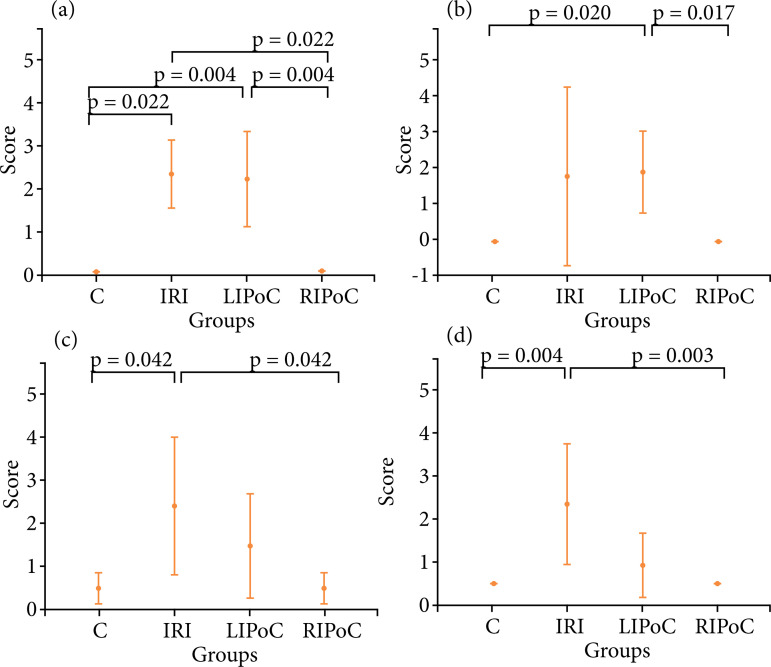
Results of histological analyses. Note the effects of IRI and the reversions promoted by LIPoC or RIPoC. **(a)** Duodenum; **(b)** Jejunum; **(c)** Proximal ileum; **(d)** Distal ileum.

**Figure 2:**
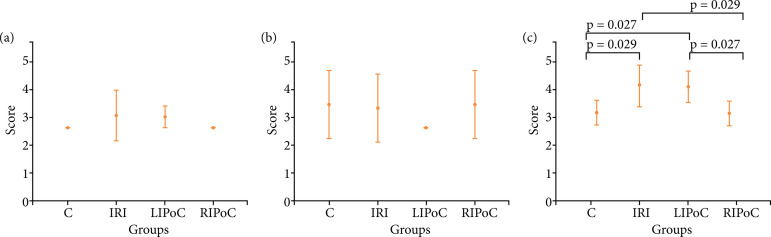
Results of histological analyses. Note the alterations in the kidney. **(a)** Liver; **(b)** Lung; **(c)** Kidney.

Histological analyses: In the duodenum, IRI promoted increased values of scores in comparison to the control group (p = 0.022 and 0.004, respectively) and these alterations were reversed by RIPoC (Fig. 1a). In the jejunum, IRI promoted alterations (p = 0.020) that could be reversed by RIPoC (Fig. 1b). In proximal and distal ileum, IRI promoted significative alterations in comparison to the control group (p = 0.042 and 0.004, respectively), and these alterations were reversed by RIPoC (Fig. 1c, d). In the kidneys, Banff scores were altered by IRI and LIPoC (p = 0.029 and 0.027, in comparison with controls, respectively) and these alterations were reversed by RIPoC. In liver and lung scores there were no differences between the groups (Fig. 2).

**Figure 3:**
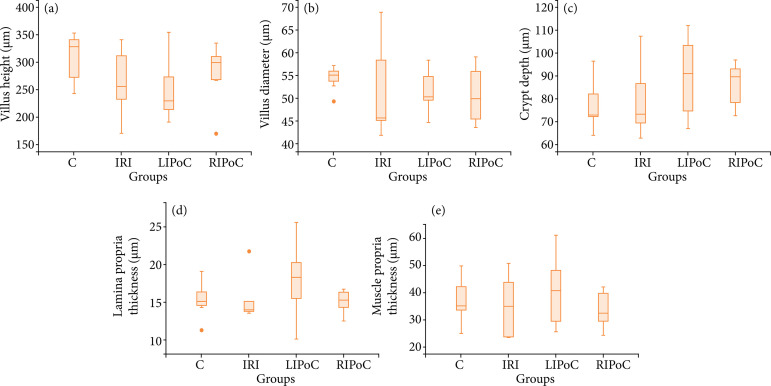
Histomorphometric analysis of duodenum. Note that there were no differences among the groups. **(a)** Villus height; **(b)** Villus diameter; **(c)** Crypt depth; **(d)** Lamina propria thickness; **(e)** Muscle layer thickness.

**Figure 4:**
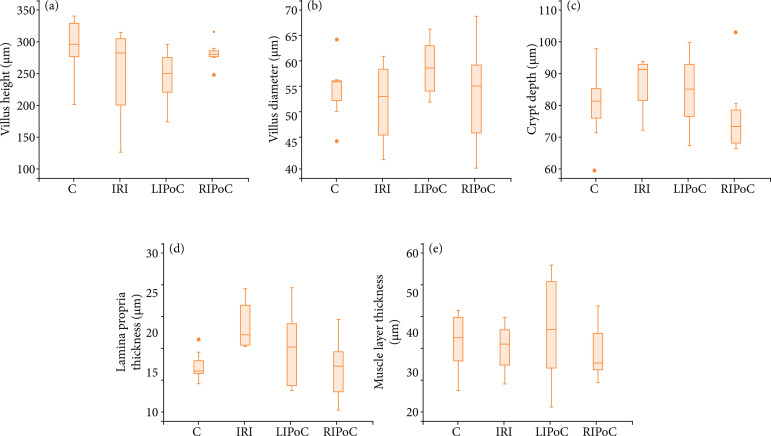
Histomorphometric analysis of jejunum. There were no differences among the groups. **(a)** Villus height **(b)** Villus diameter **(c)** Crypt depth; **(d)** Lamina propria thickness; **(e)** Muscle layer thickness.

**Figure 5:**
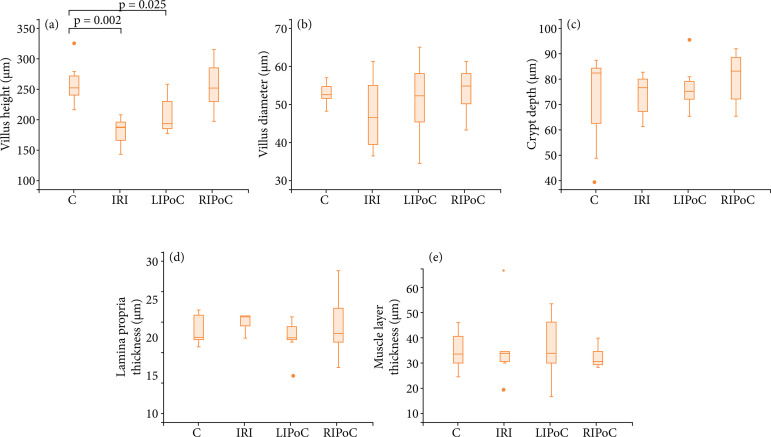
Histomorphometric analysis of proximal ileum. Note that in the proximal ileum, IRI promoted decreased values of villi height (p = 0.002) that were reversed by RIPoC. **(a)** Villus height **(b)**; Villus diameter **(c)** Crypt depth; **(d)** Lamina propria thickness; **(e)** Muscle layer thickness.

**Figure 6:**
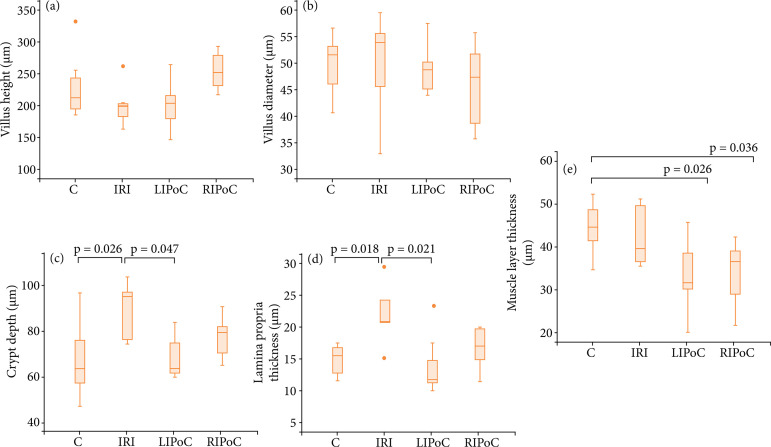
Histomorphometric analysis of distal ileum. **(a)** Villus height; **(b)** Villus diameter; **(c)** Crypt depth; **(d)** Lamina propria thickness; **(e)** Muscle layer thickness C and D – The alterations caused by IRI were reversed by LIPoC or RIPoC, except the increased muscle layer **(E)**.

The results of the histomorphometric analysis are shown in Figs. 3–6. There were no significant differences among the groups in the duodenum (Fig. 3) and the jejunum (Fig. 4). In the proximal ileum, IRI promoted decreased values of villi height (p = 0.002) that were reversed by RIPoC (Fig. 5a). In the distal ileum, the histomorphometric alterations caused by IRI could be reversed by LIPoC or RIPoC (Fig. 6c, d), except the increased muscle layer (Fig. 6e).

**Figure 7:**
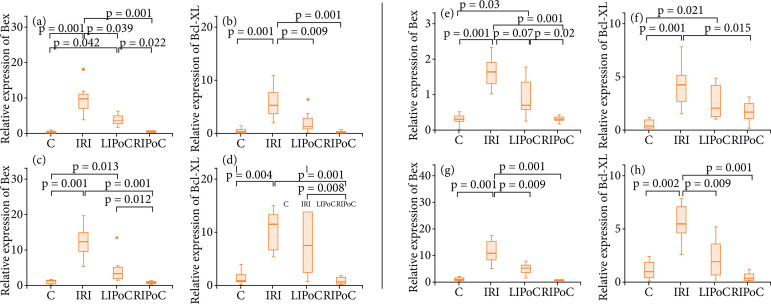
Results of gene expression studies. Note that the increased gene expressions promoted by the IRI were reversed by LIPoC or RIPoC. (a, b) Duodenum; (c, d) Jejunum; (e, f) Proximal ileum; (g, h) Distal ileum.

Gene expression analyses: Bax (proapoptotic) and BCl-XL (antiapoptotic) gene expression in duodenum, jejunum, proximal and distal ileum are expressed in Fig. 7. In general, gene expressions were increased by IRI and these alterations were equally reversed by LIPoC or RIPoC.

## Discussion

In the last 10 years, many authors have been studying the effects of ischemic preconditioning methods in small animals and pigs to find the best method to be utilized in different clinical and/or experimental situations. The final objective is liver transplantation surgery in children. So far, there is no clear evidence in the literature about the real effects of IPC in this situation. As an initial consideration, it may emphasize that the different modalities of IPC are impractical procedures that may further increase the already high complexity of transplantation surgery. This conclusion was based on a sequence of experimental investigations performed in a pig model of liver transplantation in which it was demonstrated that direct, remote ischemic preconditioning or a combination of both, produce beneficial effects only at the molecular level, without any biochemical or histological advantages^15^^,^ 16. These studies led to the conclusion that the benefits’ procedures are not consistent and may not be applied to liver transplantation in humans.

Before abandoning the studies about alleviating the harmful effects of IRI, Our group of investigators decided to try the effects of IPoC methods. Until the present time, there has been a lot of evidence that RIPoC can reduce IRI in distant organs of rats, such as the intestines^17^^-^ 19, the myocardium^20^^,^ 21, the brain^22^, and the lungs^23^. Thus, RIPoC appears to be a promising method to explore for improving IRI damage, as it is a more straightforward procedure to be utilized in the complicated surgery of liver transplantation in humans.

Regarding our experimental model and the clinical applicability of the current results, the first comment refers to the characteristics of the experimental model. The observed histological, histomorphometric, and gene expression alterations in the IRI, LIPoC, and RIPoC groups demonstrated the efficacy of the occlusion of the superior mesenteric vessels in promoting alterations comparable to those observed in liver transplantation surgery. Also, we observed that the differences among the groups are more notable at the gene expression level, secondarily at the histological level, and less evident at the histomorphometric level. This might occur because gene expression changes are more precocious and precede morphological changes at the tissue level, as we found in a previous investigation from our group^16^.

A recent similar investigation in rats conducted by Yasojima et al.^24^ demonstrated that RIPoC is the most capable technique to improve antioxidant defenses in the organism against IRI, in addition to being the most promising way to reduce the histological damage of IRI. This study stimulated us to perform the current investigation, with conclusions that match those obtained by Yasojima et al.

The histological analysis of the distal ileum showed significantly increased lesion scores for the IRI group compared to controls (crypt depth and lamina propria thickness, p = 0.026 and 0.018, respectively) that were reverted by LIPoC or RIPoC methods. This interesting finding may be explained by the increased ileum sensitivity of newborns to ischemia, as it is observed in clinical practice. In newborns with hypoperfusion states and necrotizing enterocolitis, the observed higher incidence of ileum necrosis compared to the jejunum, may corroborate the current results. Finally, it may be stressed the positive results of LIPoC or RIPoC methods by promoting the reversal of these changes.

In similar investigations performed in rats and rabbits, it was demonstrated the benefits of IPoC in reducing mesenteric ischemia-reperfusion damage. In a rat model, Chu et al.^25^ evaluated the levels of DNA fragmentation by electrophoresis, the levels of apoptosis, lipid peroxidation, superoxide production, neutrophil infiltration through malondialdehyde content, and superoxide dismutase and myeloperoxidase activities, respectively. The conclusion was that the protection of IPoC was related to reduced production of reactive oxygen species, enhanced activities of antioxidant systems, and inhibited apoptosis of intestinal mucosal cells. In a rabbit model, Yang et al.^26^ showed similar results, without any difference in the beneficial effects of LIPoC or RIPoC methods. This difference in relation to the rat model probably is due to a characteristic of the animal species. Therefore, all these conclusions corroborate the results of the current investigation.

Finally, The current investigation was refined by adding some molecular analyses that could show some beneficial effects of IPoC. It was observed a uniform response of increased expression of proapoptotic and antiapoptotic genes, Bax and Bcl-XL respectively, in response to the IRI, in the duodenum, jejunum, proximal and distal ileum. The changes were all reversed by the LIPoC and RIPoC methods, with more evident effects of RIPoC. Interestingly, we observed that RIPoC could reverse the changes to the control values, indicating the real benefits of such a procedure.

## Conclusion

We demonstrated here that local or remote postconditioning methods effectively reduce IRI in the AMI model of young rat. A particular conclusion from this investigation was that the remote method promoted more evident effects. This may contribute to understanding and better treating children with AMI. Specifically for the series of investigations conducted by the authors, the results of the current work may stimulate them to persist in investigating the best procedure to reduce the harmful effects of IRI.
